# Systematic review of microplastics and nanoplastics in indoor and outdoor air: identifying a framework and data needs for quantifying human inhalation exposures

**DOI:** 10.1038/s41370-023-00634-x

**Published:** 2024-01-06

**Authors:** Tiffany Eberhard, Gaston Casillas, Gregory M. Zarus, Dana Boyd Barr

**Affiliations:** 1 Gangarosa Department of Environmental Health, Rollins School of Public Health of Emory University, Atlanta, GA USA; 2 Agency of Toxic Substances and Disease Registry, Office of Innovation and Analytics, Atlanta, GA USA

**Keywords:** Inhalation Exposure, Air Pollution, Emerging Contaminants, Emerging Contaminants

## Abstract

**Background:**

Humans are likely exposed to microplastics (MPs) in a variety of places including indoor and outdoor air. Research to better understand how exposure to MPs correlates to health is growing. To fully understand the possible impacts of MPs on human health, it is necessary to quantify MP exposure and identify what critical data gaps exist.

**Objectives:**

The current paper provides a human exposure assessment of microplastics in the air using systematically reviewed literature that provided concentration of MPs in air as well as doses used in toxicology studies to calculate inhalation exposure dose.

**Methods:**

All published peer-reviewed journal articles, non-published papers, and grey literature that focused on micro- or nano-plastics in indoor and outdoor air were systematically searched using PRISMA guidelines. Literature that defined specific concentrations and size of MPs in air or exposed to human lung cells, animals, or humans with measurable health impacts were included in data extraction. Inhalational exposures were calculated for different age groups using published MP concentrations from the included literature using exposure dose equations and values from U.S. ATSDR and EPA.

**Results:**

Calculated mean indoor inhalational exposures from passive sampling methods were higher than those calculated from active sampling methods. When comparing indoor and outdoor sampling, calculated inhalation exposures from indoor samples were greater than those from outdoor samples. Inhalation exposures of MPs differed between age groups with infants having the highest calculated dose values for all locations followed by preschool age children, middle-school aged children, pregnant women, adolescents, and non-pregnant adults. MP doses used in toxicology studies produced higher calculated mean inhalational exposures than those from environmental samples.

**Impact:**

This study is the first known systematic review of inhalational MP exposure from indoor and outdoor air. It also provides inhalational exposures calculated from previously published environmental samples of MPs as well as from toxicology studies.

## Introduction

Plastic is an essential material and has become globally ubiquitous. Once in the environment, plastics break down into smaller pieces, a process called fragmentation [1]. Decomposed plastics are categorized based on their size, with microplastics (MPs) measuring less than 5 millimeters in diameter and nanoplastics measuring from 1 to 1000 nanometers in diameter, although in this paper we use the abbreviation MPs to collectively discuss micro- and nanoplastics [1, 2]. MPs have been found in water and soil, and recent research is exposing the vast amount of them in ambient and indoor air [3, 4]. MP exposure to humans is likely unavoidable with inhalation as one of the main routes of exposure.

After inhalation, MPs can be transported throughout the body depending on size and may end up in various organs [5]. Recent research provides evidence of MPs in human lung tissue from living people [6]. Animal models reveal potential health implications from inhaled MPs such as their transport to the brain resulting in neurotoxic effects and increased pulmonary inflammation [7, 8]. In addition, toxic plastic additives that travel with microplastics may be disrupting human health [9].

MPs have been found in indoor and outdoor air; however, even with growing concern regarding MP exposure from air, no methodological standardization for measuring MPs in the air or evaluating human exposure are available. The lack of standardization makes it difficult to compare results and findings across studies. The challenge of standardizing MP research is partly due to the recency of MPs as a substance of concern in the environment, the lack of a singular method to characterize the many MP substances, and the difficulty in obtaining and analyzing samples without contamination. The evidence of possible human health impacts from air exposure to MPs is also limited, but growing.

To our knowledge, no comprehensive exposure assessments of MPs in indoor and outdoor air exist in the current literature. This paper provides a human exposure assessment of MPs in the air via a systematic review of published peer-reviewed journal articles, non-published papers, and documents in grey literature that have provided dose or concentrations of MPs measured in indoor and/or outdoor air, human exposure to or detection of MPs in laboratory studies, and animal model experiments of exposure to or detection of MPs. Our goal is to provide a method for calculating inhalation exposure to microplastic particles and fibers in the air and a baseline for identifying factors needed to assess risk from microplastics while also identifying critical knowledge gaps that make these assessments less precise.

## Methods

### Literature review

The initial literature search research questions were: 1) what evidence exists for human exposure to MPs in the air; 2) what are the health implications for inhaled MPs; and 3) what are the gaps in research on human exposure and health due to MPs in the air? Most of the recent MP literature focuses on their presence in water, with little focus on MPs in air. As such, search terms in this review were initially kept broad to capture as many articles as possible related to MPs in the air due to the limited number of publications on the specific topic. To capture literature that may be relevant to MPs in air but may not use the term “microplastic”, search terms included common plastic polymers that are often the origin of MPs in air. These polymers included polyethylene, polyester, polyamide, polypropylene, and polystyrene. Literature that studied the aforenamed polymers with sizes <5 mm, the common size cutoff for MPs, and that fit the rest of the search criteria were included. Search terms included “Microplastic” or “Micro-plastic” or “Nanoplastic“ or “Nano-plastic” OR “Microfiber” or “Micro-fiber” or “Polyethylene” or “Polyester” or “Polyamide” or “Polypropylene” or “Polystyrene” or “(plastic ADJ5 (particulate or particle or PM), and air” or “aerosol” or “atmosphere” or “indoor environment” and “respir*” or “lung” or “inhal*” or “breath” or “asthma” or “bronch*” or “health” or “exposure” or “toxic”. There were initially no restrictions on the time of publication or language. The databases Medline, Embase, Global Health, CINAHL (EbscoHost), GreenFile (EbscoHost), Environmental Science Collection, and Scopus were searched on January 13, 2022. An additional identical literature search was completed on April 8, 2022 for the time period from January 13, 2022 to April 8, 2022. The Covidence software (Melbourne, Victoria, Australia) platform was used to manage the papers identified and review process.

All published peer-reviewed journal articles, non-published papers, and documents in grey literature that focused on MPs and their major polymer sources in indoor and outdoor air were included as well as those that focus on human exposure and routes of exposure to MPs in air and those discussing the health effects of MPs. The initial inclusion criteria for abstract screening were literature that included information about MPs in outdoor or indoor air (from now on referred to as *air*), human exposure to MPs in air, pathways of human exposure to MPs in air, health impacts/effects from exposure to MPs in air, MPs in human lung tissue and/or lung cells, respiration and/or inhalation of MPs from air, fate and transport of MPs in air, sampling and/or methods of measuring MPs in air, occupational exposure and/or occupational health impacts of MPs in air, mammal models for exposure and health outcomes from MPs from air. The definition in this review of MPs includes polymers of plastic origin that are commonly found in microplastic air samples (I.e., polyethylene, polyester, polyamide, polypropylene, and polystyrene) that are <5 mm in diameter, and only papers published in English were finally included. Literature describing PM2.5 and PM10 but with no mention of MPs and articles discussing drug delivery and clinical usage of nano-and micro technologies in any manner were excluded. Conference presentations and conference abstracts were also excluded.

Additional exclusion criteria were incorporated for full text screening. In addition to the initial criteria, full text review inclusion criteria included only literature that defined specific dose or concentrations and size and length of MPs in air, specific dose or concentration and size and length of MPs exposed to human lung cells or animals or humans with measurable health outcomes. Literature with no primary MPs exposure data and articles measuring only retention, clearance, or fate of MPs in human or animal respiratory tract or lung were excluded during full text screening. Each decision for inclusion and exclusion were decided upon by two reviewers.

Specific data were defined for extraction of the final articles for inclusion for the review. Three broad categories emerged from the included papers: Environmental sampling, Human exposure, and Animal models. Ranges and averages for dose/concentration and size of MPs as well as type and shape of plastic polymer used for exposure or detection was extracted for all papers when possible. Extracted data for literature in the environmental sampling group included sampling methods and location of sampling. Extracted data for human exposure and animal model groups included study design and health effects from MP exposure or detection.

### Human environmental exposure calculations

Multiple equations were used to determine inhalation exposures to the general population using data generated from the included literature. To determine exposure dose for active sampling that gave sampling result units in number of MPs/m^3^, the Exposure Factor Equation and Inhalation Exposure Equation were derived from Agency for Toxic Substances and Disease Registry (ATSDR) and were used to determine inhalation exposure dose from active sampling measurements [10]:

$${{{{{\rm{EF}}}}}}=({{{{{\rm{FxED}}}}}})/({{{{{\rm{AT}}}}}})$$

where EF is the exposure factor, F is the frequency of exposure (days/year), ED is the exposure duration (years), AT is the averaging time (ED x 365 days/year).

$${{{{{\rm{D}}}}}}_{{{{{\rm{inh}}}}}}=({{{{{\rm{C}}}}}} \, {{{{{\rm{x}}}}}} \, {{{{{\rm{IR}}}}}} \, {{{{{\rm{x}}}}}} \, {{{{{\rm{EF}}}}}})/({{{{{\rm{BW}}}}}})$$

where D_inh_ is the exposure dose (number of MPs/kg-BW/day), C is the contaminant concentration (MP/m^3^), IR is the intake rate (m^3^/day), and BW is the body weight (kg).

To determine inhalation exposure dose for passive deposition, a similar inhalation exposure dose equation was used for calculating daily inhalation exposures but with an added factor to convert area to volume [11, 12]:

$${{{{{\rm{D}}}}}}_{{{{{\rm{inh}}}}}}=({{{{{\rm{D}}}}}}_{{{{{\rm{r}}}}}}\times {{{{{\rm{IR}}}}}}\, \times \, {{{{{\rm{EF}}}}}})/({{{{{\rm{V}}}}}} \, {{{{{\rm{x}}}}}} \, {{{{{\rm{BW}}}}}})$$

Where D_inh_ is inhalation exposure dose (number of MPs/kg-BW/day), D_r_ is the deposition rate (MPs/m^2^/day), IR is the intake rate (m^3^/day), EF is the exposure factor (unitless and calculated in the previous equation), BW is the body weight (kg), and V represents the volume of air (m^3^) of a 1 m^2^ sampling area. V was determined by subtracting the sampling height from the standard height of indoor places (2.4 m) [11, 12].

Inhalation exposures were calculated separately for six age groups commonly used in exposure calculations: infant (birth to <1 year), preschooler (2 to <6 years), middle childhood/young children (6 to <11 years), adolescent (11 to <16 years), pregnancy (second trimester), and (adult ≥ 21 years). Variables specific to each age group for EF as well as IR and BW were derived from ATSDR for all age groups except pregnant women where EPA exposure standards were used for the second trimester [10, 13–16]. Calculations were done with all groups being exposed to the same levels of MPs per sampling location. These calculations focused on determining the estimated exposures to the general populations for each group.

Ten location groups were established based on descriptions from included papers (*residential*, *workplace*, *school*, *infrequent*, *indoor combined*, *outdoor urban*, *outdoor remote*, *roadside*, *occupational*, and *rooftop*). *Residential* includes samples collected in any indoor living space in houses or apartments. *Workplace* sampling locations included any indoor work locations, offices, hallways, reception areas, and conference rooms. *School* sampling locations include kindergarten through high school settings and university classrooms. *Infrequent* category includes samples taken inside a nail salon, hospital, and mosque. While those who work or spend greater amounts of time within these areas will have continuous exposures, the general population will be infrequently exposed. *Indoor combined* values represent a daily average exposure because they are the sum of the exposures obtained from multiple locations throughout the day and include residential, workplace, school, healthcare facilities, and public transit halls. *Outdoor urban* includes samples collected outside in urban areas such as town centers, shopping areas, and urban residential streets. *Outdoor remote* samples were collected outside in remote areas such as forests and farmland. *Roadside* samples were collected along roads in urban and industrial areas. *Occupational* samples were collected in a waste transfer station and plastic recycling facility during injection molding steps and grinding of plastic. *Rooftop* samples were collected on the roof of buildings between 3 and 38 meters above ground level. Populations that work or otherwise spend more time in any of the areas that have higher concentrations of MP will have even higher exposures.

*Residential* EF was assumed to be 1 for all age groups (24 h/day, 365 days/year) [14]. *Outdoor urban* and *roadsides* EFs were also assumed to be 1 due to potential daily exposure. The average adult lifetime of 78 years was used to calculate adult ED of 57 years for all categories where lifetime exposure was assumed. The only location category where adult ED was less than 57 years was for *workplace* exposure duration which was calculated based on full retirement of age 67 years which equated to 46 years for adult *workplace* exposure [17]. Pregnancy ED was assumed for nine months total (0.75 years). For *workplace* frequency of exposure (F), full time exposure of 50 weeks/year was used [17]. The central tendency estimate (CTE) was used for values for infants at childcare facilities and preschoolers (school location category) which is 50 wks/year. CTE values for middle childhood and adolescent school times are 39 wks/year [10]. Frequency of exposure (F) for adolescent age group and younger were given a zero for *workplace* location. Adults and pregnant women were given a zero for frequency of exposure (F) for *school* location. However, it is recognized that some pregnant woman, parents, and teachers will spend time in schools, yet the general population likely does not. *Outdoor remote* locations were assumed to have two days per month of exposure (F) and *infrequent locations* and *rooftop* sampling was assumed to have one day per week exposure (F).

### Human and animal toxicology studies

Human and animal exposure studies were analyzed for dose and concentration of MPs to use for calculating estimated exposures in number of MPs/kg-BW/day to compare with environmental exposure data. MP exposure data from literature that provided mass per volume (mg/L or μg/L) units were used to calculate exposures in number of particles or fibers per volume (MPs/m^3^) using the conversion equations from Leusch and Ziajahromi where x is the concentration of MP beads in particles/L units or concentration of microfibers in fibers/L units and y is the concentration of MP beads or fibers in mg/L units [18]:

$${{{{{\rm{x}}}}}}({{{{{\rm{beads}}}}}}/{{{{{\rm{L}}}}}})=\frac{{{{{{\rm{y}}}}}}\left(\frac{{{{{{\rm{mg}}}}}}}{{{{{{\rm{L}}}}}}}\right)\times {10}^{9}\left({{{{{\rm{unit}}}}}}\; {{{{{\rm{conversion}}}}}}\; {{{{{\rm{factor}}}}}}\right)}{\left(\frac{\Pi }{6}\right)\times {{{{{\rm{density}}}}}}\left(\frac{{{{{{\rm{g}}}}}}}{{{cm}}^{3}}\right)\times {\left[{{{{{\rm{diameter}}}}}}\left({{{{{\rm{\mu }}}}}}{{{{{\rm{m}}}}}}\right)\right]}^{3}}$$

$${{{{{\rm{x}}}}}}({{{{{\rm{fibres}}}}}}/{{{{{\rm{L}}}}}})=\frac{{{{{{\rm{y}}}}}}\left(\frac{{{{{{\rm{mg}}}}}}}{{{{{{\rm{L}}}}}}}\right)\times {10}^{9}\left({{{{{\rm{unit}}}}}}\; {{{{{\rm{conversion}}}}}}\; {{{{{\rm{factor}}}}}}\right)}{\Pi \times {\left[{{{{{\rm{radius}}}}}}\left({{{{{\rm{\mu }}}}}}{{{{{\rm{m}}}}}}\right)\right]}^{2}\times {{{{{\rm{length}}}}}}\left({{{{{\rm{\mu }}}}}}{{{{{\rm{m}}}}}}\right)\times {{{{{\rm{density}}}}}}\left(\frac{{{{{{\rm{g}}}}}}}{{{cm}}^{3}}\right)\,}$$

The densities used for polystyrene, PET, and polyester were 1.05 g/cm^3^, 1.397 g/cm^3^, 1.37 g/cm^3^, respectively [18, 19].

Since toxicological doses are much higher and particle size is much lower than those typically found in current environmental samples, the largest sized MP and the minimum dose of MPs in each study were used for calculations to determine the estimated exposure dose in number of MPs/kg-BW/day for human and animal toxicology studies to try and best align with MP environmental sampling results.

## Results

### Literature review

**Fig. 1:**
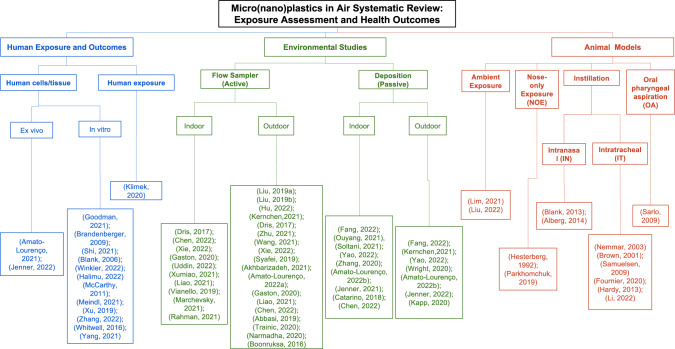
Preferred Reporting Items for Systematic Reviews and Meta-Analysis (PRISMA) diagram of review process. (*n*) represents the number of studies.

A total of 7587 articles were found in the initial search on January 13, 2022. After removing duplicates, 4863 articles were remaining for abstract screening. From the second literature search from January 13, 2022 to April 8, 2022, an additional 268 articles were found with 115 remaining after duplicates were removed totaling 4978 abstracts screened. After screening at the abstract level, 258 articles were included for full text review. The scope of inclusion criteria for full text review was limited to papers that gave dose or concentration of MPs in air. After screening full text literature, 61 papers were included for data extraction separated into three categories based off of data for exposure calculations: Human Exposure and Outcomes [6, 20–33], Environmental Studies [6, 12, 34–65], and Animal Models [7, 8, 66–76] (Fig. 1).

Environmental sampling papers were subdivided into active flow sampling (*n* = 23 papers) and passive deposition (*n* = 13 papers) sample collection methods with 2 papers having both active and passive sampling totaling 34 papers in the environmental sampling group.

**Fig. 2:**
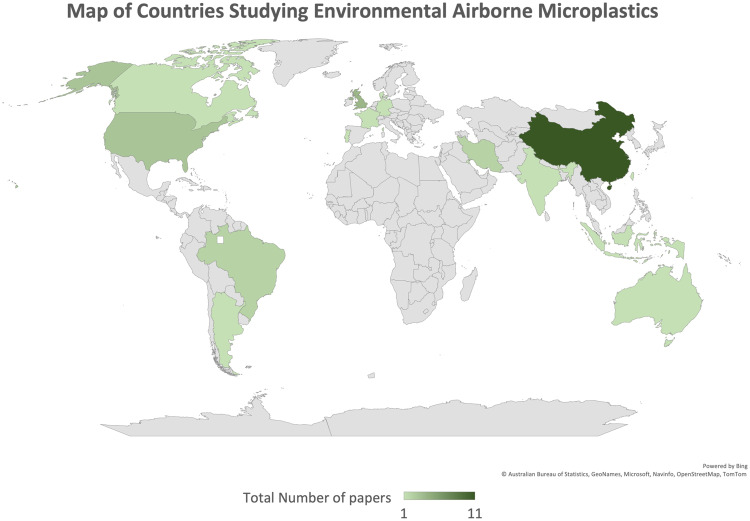
World Map of countries included in this systematic review with published papers measuring MPs in Air.

Environmental sampling papers were analyzed based on country of MP sampling with China publishing the most papers on MP in air captured in our systematic review (*n* = 11 papers), followed by the United Kingdom (*n* = 4 papers), the United States (*n* = 3 papers), Iran and Brazil (*n* = 2 papers each), and the remaining countries publishing 1 paper (Fig. 2).

**Fig. 3:**
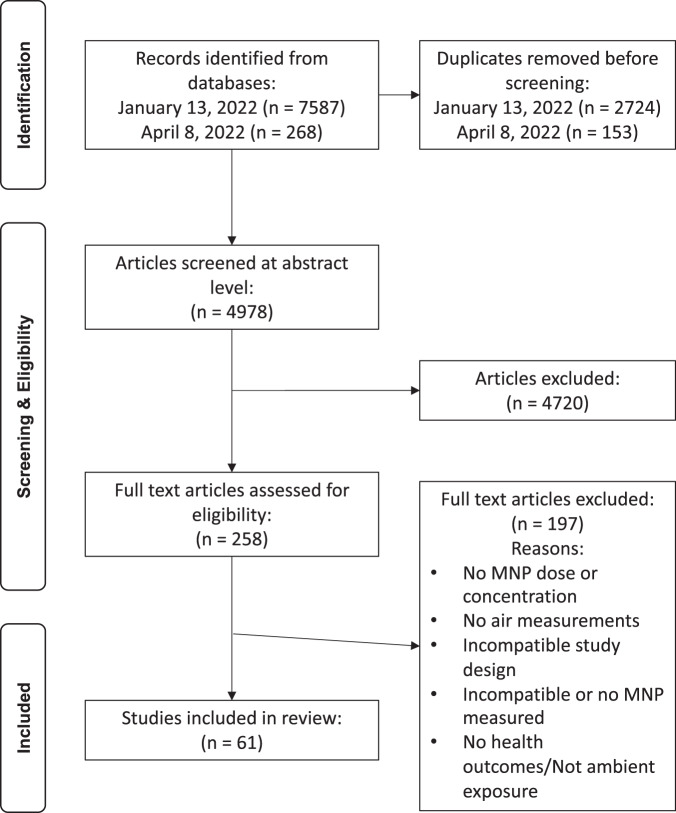
Flow Diagram of included articles for each group.

Literature in the human toxicology category included in vitro studies using human lung cells (*n* = 12 papers), ex vivo articles measuring MPs in human lung tissue with one article included in the environmental sampling papers as well [6] (*n* = 2 papers) and human exposure to MPs (*n* = 1 paper) totaling 15 papers in the human toxicology grouping. Literature defined in the animal toxicology group was subdivided based on method of exposure and included instillation of MPs either intratracheally (*n* = 6 papers) or intranasally (*n* = 2 papers), and ambient exposure (*n* = 2 papers), nose-only exposure (*n* = 2 papers), and oral pharyngeal aspiration (*n* = 1 paper) of MPs to animals totaling 13 papers in the animal exposure group (Fig. 3).

### Human environmental exposure calculations

Data generated from included papers measuring environmental indoor and outdoor MPs in air were in multiple units of measurement depending on the methods of sample collection and analysis. Active sampling of MPs using a flow sampler had units of measurement in number or unit of MP per volume of air (MP/m^3^) and were most often collected as particles, fibers, fragments, or a combination of different shapes of MPs. Passive sampling, often referred to as deposition of MPs, provided units in number of MPs per area (MPs/m^2^/day).

### Sampling location results

Locations of environmental MP air sampling were categorized into nine location groups: *residential* (*n* = 8), *workplace* (*n* = 7), *school* (*n* = 2), *infrequent* (*n* = 3), *indoor combined* (*n* = 3), *outdoor urban* (*n* = 8), *outdoor remote* (*n* = 4), *roadsides* (*n* = 3), *occupational* (*n* = 3) and *rooftops* (*n* = 7) for a total of 48 sample locations. Of these, 13 sampling locations were removed prior to analysis due to incompatible measurement units (total fiber count, no specific MP values given, only ranges of concentrations, historically collected data, and high values skewing the data).

For calculations of inhalation exposure dose, 29 sampling location values were used from active sampling which included *residential* (*n* = 4), *workplace* (*n* = 2), *infrequent* (*n* = 3), *indoor combined* (*n* = 2), *outdoor urban* (*n* = 7), *outdoor remote* (*n* = 3), *roadsides* (*n* = 1), *occupational* (*n* = 3), and *rooftops* (*n* = 4). For passive deposition, 19 sampling location values were collected (*n* = 12 indoor and *n* = 7 outdoor samples). Outdoor samples collected via passive deposition were removed prior to analysis; only indoor papers were used for passive deposition inhalation exposure dose calculations due to the conversion of area to volume. A total of 10 sampling locations were used in final inhalation exposure dose calculations from passive sampling with 2 samples not reporting sampling height and therefore not able to be used to calculate area to volume for use in exposure dose equations. The included samples for passive deposition were from these locations: *indoor combined* (*n* = 1), *residential* (*n* = 4), *school* (*n* = 1), and *workplace* (*n* = 4).

### Calculations by age and location

**Fig. 4:**
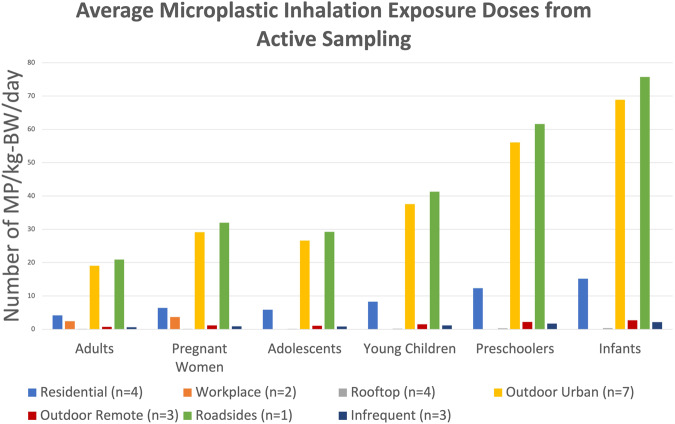
Active sampling average inhalation exposures for age categories and locations of sampling for indoor and outdoor where n is the number of sampling locations used for the average.

Infants had the highest calculated inhalation exposure dose values for all locations followed by preschool age children, middle aged children, pregnant women, adolescents, and finally adults. Average inhalation exposures for active sampling can be found in Table 1. Roadsides had the highest calculated exposure dose of airborne MPs using active sampling after indoor combined locations (Fig. 4).

**Table 1:** Average MP exposure doses from active sampling for different locations by age groups (MPs/kg-BW/day) and (*n*) represents the number of studies.

Location of Samples	Adults (≥ 21 years)	Pregnant Women (second trimester)	Adolescents (11 to < 16 years)	Young Children (6 to < 11 years)	Preschoolers (2 to < 6 years)	Infants (birth to < 1 year)
**Indoor Combined (*n*** = **2)**	152	232	213	300	447	550
**Residential (*n*** = **4)**	4.19	6.41	5.86	8.27	12.3	15.2
**Workplace (*n*** = **2)**	2.39	3.66	0	0	0	0
**Rooftop (*n*** = **4)**	0.094	0.143	0.131	0.184	0.275	0.338
**Outdoor Urban (*n*** = **7)**	19	29.1	26.6	37.6	56.1	68.9
**Outdoor Remote (*n*** = **3)**	0.742	1.14	1.04	1.46	2.19	2.69
**Roadsides (*n*** = **1)**	20.9	32	29.3	41.3	61.6	75.7
**Infrequent (*n*** = **3)**	0.585	0.896	0.819	1.15	1.72	2.12

Roadside exposure calculations were taken from one study by averaging sample values taken from roadsides with low, medium, and high traffic; results showed that larger numbers of MPs were found in roadsides with higher traffic volumes [50]. The average of calculated outdoor urban exposures followed the same trend for age groups at exposure dose levels similar to roadsides. For indoor sampling, residential locations had the highest calculated MP exposures ranging from calculations for one sample at 0.21 MPs/kg-BW/day for adults to 43.35 MPs/kg-BW/day from one sample for infants. Calculated workplace MP inhalation exposures were calculated from two studies with adult doses ranging between 1.17 to 3.61 MPs/kg-BW/day and pregnant women ranging between 1.79 to 5.52 MPs/kg-BW/day [34, 38]. Outdoor remote location exposures were calculated to be less than 5 MPs/kg-BW/day for all sampling locations in every age group, with the lowest exposure dose being 4.9 × 10^−5^ for an adult at one outdoor remote location. Calculated rooftop exposures were all at or under 1 MPs/kg-BW/day. Indoor combined MP inhalation exposures were calculated to be the highest out of all sampling locations, which is understandable given that indoor combined included environmental MP samples averaged among various day-to-day locations which provide a good representation of daily exposure. Calculated averages in this location group ranged from 151.9 MPs/kg-BW/day for an adult to 549.8 MPs/kg-BW/day for an infant.

**Fig. 5:**
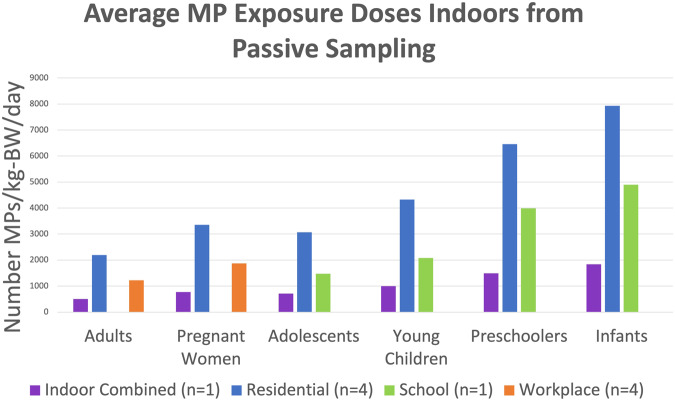
Passive deposition sampling average inhalation exposures for age categories and locations of sampling for indoor environments where *n* is the number of sampling locations used for the average.

**Fig. 6:**
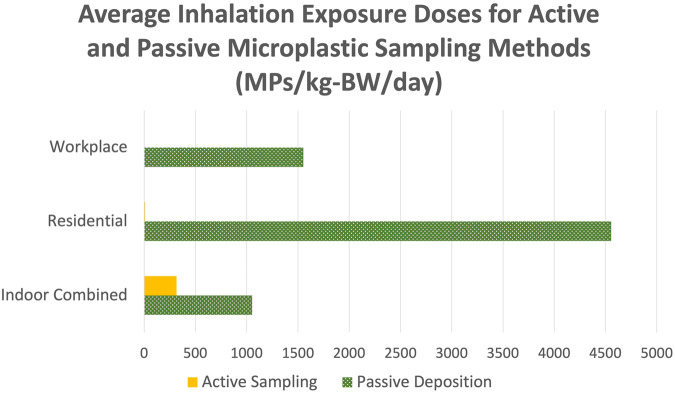
Comparison of average inhalation exposures between active sampling (*n* = 2, 4, and 2 sampling locations for indoor combined, residential, and workplace, respectively) and passive deposition (*n* = 1, 4, and 4 sampling locations for indoor combined, residential, and workplace, respectively) of MPs at three indoor locations.

**Fig. 7:**
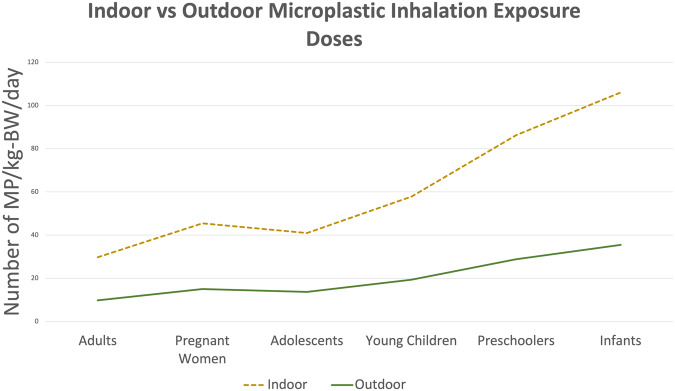
Comparison of Indoor and Outdoor MP inhalation exposures from active sampling among different age groups.

Average inhalation exposure for passive deposition sampling can be found in Table 2. As stated previously, only indoor sampling locations were used for calculated inhalation exposures (Fig. 5). While the trend of age group differences between exposures may be similar, the inhalation exposures for passive deposition sampling are higher than for active sampling (Fig. 6). The calculated average *indoor combined*, *residential*, and *workplace* inhalation exposures for passive deposition from all age groups is 1053, 4555, and 1552 MPs/kg-BW/day, respectively. This is compared with active sampling in which the average inhalation exposures for *indoor combined*, *residential*, and *workplace* is 315, 8.71, and 3.02 MPs/kg-BW/day, respectively. When comparing indoor to outdoor MP inhalation exposures for active sampling, indoor average exposures are higher for all age groups compared with outdoor doses (Fig. 7).

**Table 2:** Average MP exposure doses from passive sampling for different locations (indoors) by age groups (MPs/kg-BW/day) and (*n*) represents the number of studies.

Location of Samples	Adults (≥ 21 years)	Pregnant Women (second trimester)	Adolescents (11 to < 16 years)	Young Children (6 to < 11 years)	Preschoolers (2 to < 6 years)	Infants (birth to < 1 year)
**Indoor Combined (*n*** = **1)**	507	775	709	1000	1493	1835
**Residential (*n*** = **4)**	2192	3354	3068	4326	6456	7936
**School (*n*** = **1)**	0	0	1477	2083	3986	4900
**Workplace (*n*** = **4)**	1227	1877	0	0	0	0

### Occupational sampling results

Two occupational studies shed light on human exposures to airborne microplastics. Boonruska et al. describes nanoparticles and fibers in the air showing airborne concentrations of polypropylene particles during manufacturing and recycling of carbon nanotubes reinforced with polypropylene composites ranging from 1.2 ×10^3 ^cm^−3^ to 4.3 ×10^5^ cm^−3^ [57]. Using these environmental samples from the air, we calculated inhalation exposures to be 535 MPs/kg-BW/day for injection molding steps and 22531 MPs/kg-BW/day during the grinding stage of recycling of polypropylene. Hu et al. describes microplastics emissions through a roof vent in a municipal waste setting with an average concentration of 2.5 + /− 1.3 MPs/m^3^ microplastics [46].

### Human and animal toxicology studies

Of the 15 human exposure studies included in data extraction, 24 concentrations of MPs were identified as applied to cells or detected in human lung tissue. Of these, 8 concentrations were removed because of incompatible units used to determine concentrations, detected MPs, or insufficient information for the calculations from mass per volume to number per volume of MPs. A total of 16 MP concentrations were calculated and converted to number of MPs per m^3^ and subsequently to inhalation exposure dose of number of MPs/kg-BW/day.

The largest sized MPs and the smallest dose of MPs from each experiment were used for calculations to exposure dose to try to best mimic environmental sampling results. The average number of MPs/m^3^ calculated from the human toxicology studies was 2.14 × 10^17^ MPs/m^3^ with ranges between 1.02 × 10^5^ to 2.27 × 10^18^ and a median value of 2.09 × 10^15^ MPs/m^3^. The adult average exposure dose calculated from the MPs/m^3^ values was 5.73 × 10^16^ MPs/kg-BW/day with a range from 2.73 × 10^4^ to 6.08 × 10^17^ MPs/kg-BW/day and median value of 5.59 × 10^14^ MPs/kg-BW/day.

There were 13 papers and 13 different concentrations from the animal model exposure group. After removing doses in incompatible units or with insufficient information for exposure dose calculations, 7 papers with 7 concentrations of MPs remained for analysis. Out of the 7 MP concentrations, the number of MPs/m^3^ was calculated and averaged to be 1.94 × 10^16^ MPs/m^3^ with ranges from 6.8 × 10^6^ to 7.58 × 10^16^ MPs/m^3^ and a median value of 9.09 × 10^13^ MPs/m^3^. The adult average MP inhalation exposure dose was calculated from the MPs/m^3^ values and gave an average of 5.18 × 10^15^ MPs/kg-BW/day ranging from 1.82 × 10^6^ to 2.03 × 10^16^ MPs/kg-BW/day and median value of 2.43 × 10^13^ MPs/m^3^. All relevant data can be found in the Supplementary Information.

## Discussion

This systematic review provides a baseline for conducting additional research on MP in the air and human exposure assessments. Our calculations relied on set values from the U.S. ATSDR and EPA as well as doses and concentrations of MPs from previously published papers; the accuracy of the calculated inhalation exposures are limited to these values and standards and may not reflect the true inhalation exposures of the set populations and locations. Our results are intended as estimates for the inhalation exposures of MPs to the general population in broadly defined locations and are not intended for personal exposure estimates in specific locations. As such, our findings suggest that sampling methods, location, and age group of exposed population to MPs in air may impact the estimated inhalation exposure dose values.

### Literature review

MP research is still in its infancy, so no universal standards currently exist. Many published papers have different sampling methods, quality control, and analyses. We did not exclude papers based on the aforementioned differences which may have resulted in the inclusion of papers that used different research methods. In addition, the specific polymers used in our systematic review search (polyethylene, polyester, polyamide, polypropylene, and polystyrene) are not exhaustive of the many different types of source material of MPs; future research on other polymer sources of airborne MP would be beneficial.

### Human environmental exposure calculations

The two main methods of sample collection for MPs in indoor and outdoor air are active sampling and passive deposition. Active sampling uses pumps to sample a known volume of air for a set time period with most studies providing units of measurement in number of MPs per m^3^ [61]. Passive deposition has been a common method of sampling atmospheric MPs and recently, progress has been made to standardize collection using metallic or glass dishes with protocols designed by NILU (Norwegian Institute for Air Research) [61]. In the current review, papers measuring MPs using active sampling accounted for about 63% of included papers in the environmental sampling group and papers with methods using passive deposition were about 37%. When comparing calculated inhalation exposures of MPs between active and passive sampling methods, passive sampling papers had higher levels of inhalation exposures for all comparable locations than active sampling. The length of sampling time differed between active and passive sampling as well. Most active sampling methods had pumps pulling air for under 24 hours with varying degrees of flow rates and volume of air sampled. Passive sampling methods varied, with researchers leaving deposition equipment open for 24 hours (*n* = 2 papers), 1 to 4 weeks (*n* = 8 papers), and 6 months or more (*n* = 2 papers).

Exposure values for rooftop samples were analyzed with the understanding that most people spend little time in this environment; however, rooftop samples may give us insight into MP atmospheric transport including deposition, contaminant transport over long distances, as well as possible exposure in high-rise apartments or buildings with windows open and/or terraces. A recent study found that MP concentrations were positively associated with PM_2.5_ and polycyclic aromatic hydrocarbons (PAHs) on a three-meter-high building rooftop and were significantly higher on dusty days compared to normal days [51]. In addition, airborne MPs have been found in remote areas such as on mountain tops, wetlands, and in the middle of the North Atlantic Ocean, [40, 55, 77] suggesting distant MPs atmospheric transport and possible human exposure in areas where the MPs did not originate.

Pregnant women show higher calculated inhalation dose exposure values compared to averages for adults. One possible explanation for the differences in exposures is due to higher inhalation rates during pregnancy which changed the IR variable for each group in the equations used to calculate inhalation exposure dose. Another factor to consider when comparing pregnancy exposure data to adult data is that adult exposure dose is averaged for both males and females combined, with slightly higher variables for combined data than would be obtained for disaggregated data by sex. Even so, pregnant women and developing babies are more susceptible to toxins, especially long-term health complications from exposure in utero [78]. MPs have been detected in human placental tissue and meconium [79], suggesting maternal and fetal exposure to MPs. Animal models show inhaled MP transport from mother to fetal liver, heart, lung, kidney, and brain and have been associated with reduced fetal weight after maternal pulmonary exposure to nanoparticles [73]. During additional times of rapid development such as infancy and childhood, there may also be a greater risk for health impacts from exogenous toxins carried on the MP [78]. Our data suggest that school may be a source for MP inhalation exposure, with MP exposures for infants, young children, and adolescents higher than for adults at the workplace (Fig. 5).

Our results agree with previous studies showing that MPs have on average higher concentrations in indoor than outdoor air [36, 40, 53, 58]. Indoor dust concentrations and low air circulation could be contributing factors for the disparity between indoor and outdoor MPs. One study identified MP accumulation on air conditioning filters and measured MPs released into indoor air when the AC was on, although it was only a small percentage of the total MP concentration [80]. Another study found significantly higher airborne MP concentrations when the air conditioning unit was on for all lengths of time studied compared to when it was turned off [61]. The same study analyzed MPs in the air on weekdays versus weekends and found that in a university dormitory room, the MPs were threefold higher on weekends than on weekdays [61].

The calculated MP exposures from the two occupational studies (three sampling locations) in our analysis were much higher than the other locations analyzed and therefore were not included in our figures or discussion of exposure to the general population [46, 57]. The nature of occupational studies makes them difficult to extrapolate to exposures for the general population. Additional occupational data on airborne environmental MP should be collected to better inform workers of their exposure to airborne MPs.

While our data show roadside exposure as the highest average inhalation exposure dose for all age groups, the outdoor remote and rooftop exposures are much lower than all other location groups bringing down outdoor exposure dose averages. In addition, more dense roadside traffic was found to increase the number of airborne MPs [50] as well as urban air MP abundance being about 2x greater than rural areas in one study [40]. Therefore, while average outdoor inhalation exposures are lower than the average exposures indoors, it seems to be highly dependent on specific locations and behavior patterns. In addition, the variables used for inhalation exposure calculations are based on US population averages which may not represent the global population. For example, Kashfi et al. estimated expose doses using lower body weight and inhalation rate for all age groups than in this paper, most likely due to differences in population averages [11]. These differences may lead to different inhalation exposure dose calculations for papers using data from various countries.

Size of MPs plays a role in exposure and affects health impacts, with smaller sized particles and fibers depositing deeper in the lung and throughout the body [5]. Current sampling and analysis methods identify plastic particles in the micrometer range; however, as MP size decreases in air, some studies find that concentration increases [34, 40, 58, 80]. One study identified about three times higher concentrations of particles in the inhalable fraction than respirable fraction [34]. It is possible that we underestimated total MP exposure because the current measurements do not include smaller particles that may exist in the air. Typically, atmospheric particulates are found to follow normal distributions depending on their source, with heavier and typically larger particles falling more quickly than smaller ones [81]. Typical atmospheric dust may have a similar number of particles near 0.5 microns (in diameter) as there are at 20 microns [82]. As no studies of atmospheric microplastics are available to discern distributions at the lower levels, it is possible that they follow the trends of other soft minerals which more easily erode to smaller sizes. Particle size also affects concentrations, especially in dosing for toxicology studies. For example, Goodman et al. calculated that 10 micron MPs require a concentration of 50.0 μg/ml to match the concentration in particles/mL of 1 micron MPs at 0.05 μg/ml [21]. Environmental MP samples contain a mixture of sizes and shapes, and it is still unknown to what extent humans are exposed to nanoplastics.

### Human and animal toxicology studies

We calculated the inhalation exposure dose values for toxicology studies done in vitro, in vivo, and with ambient exposure using the doses of MPs given to live animals or human in vitro cells to enable a comparison of doses used in toxicology studies to MP doses found in MP environmental samples from air. We compared the calculated toxicological exposure dose values to the calculated exposure dose values from environmental MP samples from the systematic review. Our results suggest that MP doses given to animals and in vitro are higher than MP doses measured in air with the lowest dose and largest size of MPs used in toxicology studies higher than all calculated exposures from environmental samples except the occupational samples. The lowest estimated MP exposure dose calculated for an adult in this paper from in vitro human cell studies was 2.73 × 10^4^ MPs/kg-BW/day and the lowest estimated MP exposure dose for animal studies was 1.82 × 10^6^ MPs/kg-BW/day. The highest exposure dose calculated from environmental samples from air for an adult was 3.14 × 10^3^ MPs/kg-BW/day for a passive sample, which is still lower than the lowest exposures used in toxicology studies.

Most studies included in this review for toxicology experiments (12 out 16 human in vitro studies and 6 out of 7 animal studies) are exposing cells and animals to nanoparticles. However, at the current time most articles identified only particles in the micron size range in the air so a direct comparison may not be feasible. In addition, since exposure dose units are in number of particles per weight, the values may be higher given nanoparticles are 1000x smaller than microparticles which would give higher exposures when calculating number of particles from weight. However, these data could be beneficial for future work when sampling methods are able to more accurately measure nanoparticles in the environment. With most toxicological studies using plastic particles in the nanoscale and dosing using volumetric concentrations, it is important to account for size and shape of exposed particles in toxicology research to determine our exposure to plastics in the nano range to inform policy and future research. This study did not analyze the various sized particles collected; however, it is important to understand the size ranges of MP exposure to estimate fate and transport within the body and health implications.

### Data gaps

While the published literature clearly demonstrates that humans are exposed to MPs in air, the exposure and health implications have not yet been fully characterized. This systematic review includes publications measuring dose or concentration of MPs in air and in human and animal toxicology studies. From the articles meeting our inclusion criteria, we calculated the inhalation exposure dose for MPs for varying age groups and exposure locations. This paper collected and collated information in one source document to provide a framework for calculating exposure dose of MPs as a step toward calculating risk from varying MP mixtures that humans are exposed to; this review also acts as a springboard to continue to fill in the data gaps around human inhalation exposure to MPs. The data gaps we identified for calculating human exposure to MP in the air include, but are not limited to:

1. Current sampling methods mainly identify plastic particles in the micrometer range and may not capture smaller particles that exist in the air. This could lead to an underestimation of total MP exposure.
2. Additionally, these methods have not fully demonstrated that they capture all particle sizes with equal efficiently for all synthetics. Chamber studies to study capture efficiency for varying particle sizes of several synthetics are recommended.
3. The health impacts of MPs are influenced by particle size and bioavailability, which has implications on deposition in the lungs and distribution throughout the body. Yet, the current measurements do not include the smaller particles that may exist in the air and studies on bioavailability are limited.
4. MP research is still emerging with no universal standards, leading to varied sampling methods, quality control measures, and analyses across different studies. This has implications on the quality and comparability of data.
5. The specific polymers targeted in this review are not exhaustive of the different types of source material of MPs [4, 83]. This leaves a gap for future research on other or specific polymer sources of airborne MPs.

### Disclaimer

The findings and conclusions in this report are those of the authors and do not necessarily represent the official position of the Centers for Disease Control and Prevention, the Agency for Toxic Substances and Disease Registry, and the National Center for Environmental Health. The use of product names in this presentation does not constitute an endorsement of any manufacturer’s product.

### Supplementary information

Supplementary information
